# MicroRNA–Gene Interactions Impacted by Toxic Metal(oid)s during EMT and Carcinogenesis

**DOI:** 10.3390/cancers14235818

**Published:** 2022-11-25

**Authors:** Franklin Tran, Eunji Lee, Suresh Cuddapah, Byeong Hyeok Choi, Wei Dai

**Affiliations:** Division of Environmental Medicine, Department of Medicine, Grossman School of Medicine, New York University, New York, NY 10010, USA

**Keywords:** metals, arsenic, cadmium, chromium, nickel, microRNAs, EMT, carcinogenesis

## Abstract

**Simple Summary:**

Epithelial–mesenchymal transition is characterized by the loss of cellular adhesion and an increase in cellular motility. This phenomenon is mediated through several oncogenic signaling pathways. MicroRNAs are epigenetic regulators that can modulate both oncogenic signaling pathways and the expression of cellular adhesion proteins. Many toxic metal(loid)s are known human carcinogens and can modulate microRNA expression, resulting in epithelial–mesenchymal transition and carcinogenesis. This mini review summarizes the microRNA–gene interactions of toxic metal(loid)s in epithelial–mesenchymal transition and carcinogenesis. By doing so, we hope to highlight certain miRNAs that can be potential therapeutic targets in treating metal carcinogenesis. We also present original research findings that further characterize the mechanisms of cadmium-induced epithelial–mesenchymal transition.

**Abstract:**

Chronic environmental exposure to toxic metal(loid)s significantly contributes to human cancer development and progression. It is estimated that approximately 90% of cancer deaths are a result of metastasis of malignant cells, which is initiated by epithelial–mesenchymal transition (EMT) during early carcinogenesis. EMT is regulated by many families of genes and microRNAs (miRNAs) that control signaling pathways for cell survival, death, and/or differentiation. Recent mechanistic studies have shown that toxic metal(loid)s alter the expression of miRNAs responsible for regulating the expression of genes involved in EMT. Altered miRNA expressions have the potential to be biomarkers for predicting survival and responses to treatment in cancers. Significantly, miRNAs can be developed as therapeutic targets for cancer patients in the clinic. In this mini review, we summarize key findings from recent studies that highlight chemical–miRNA–gene interactions leading to the perturbation of EMT after exposure to toxic metal(loid)s including arsenic, cadmium, nickel, and chromium.

## 1. Introduction

Epithelial–mesenchymal transition (EMT) is a biological process in which epithelial cells lose cellular polarity and tight junctions but gain migratory and invasive characteristics. EMT is essential in cell–cell and cell–matrix adhesion, cell polarity, development, wound healing, and tissue homeostasis [1,2,3,4,5]. During EMT, epithelial cells reorganize their cytoskeleton and change gene expression profiles, leading to an increase in motility of individual cells and gaining of an invasive phenotype. However, neoplastic epithelial cells have gained the ability to deregulate EMT to promote cancer motility and invasiveness [6].

Epithelial and mesenchymal cells each express distinct gene products that contribute to adhesion and/or migration. ZO-1, occludin, E-cadherin, desmoplakin, and cytokeratins are highly expressed in epithelial cells [7,8], whereas Snail, Twist, N-cadherin, ZEB1, vimentin, and Claudin-1 are exclusively expressed, or highly enriched, in mesenchymal cells [9,10]. In normal development, EMT targets the cellular adhesion machinery by downregulating epithelial markers that make up tight junctions, zonula adherins, and desmosomes [11,12]. Among epithelial biomarkers, E-cadherin is well-characterized and functions as an important tumor suppressor, as it prevents uncontrolled cell division outside of the epithelium [13]. The loss of E-cadherin is an important initiation step in EMT. Matrix metalloproteinases (MMPs) then degrade the extracellular matrix and allow cancer cells to migrate away from their host tissue [14]. Induction of mesenchymal markers, such as N-cadherin, α-SMA, and vimentin enables tumor cells to interact with endothelial cells and enter the bloodstream to initiate metastasis [15].

Transforming growth factor beta (TGF-β) is the master regulator of EMT and is overexpressed in many cancer types. TGF-β signaling plays an important role in the translocation of Smad complexes into the nucleus to promote or repress TGF-β target genes [16]. Snail1, Snail2, Zeb-1, and SIP-1 are genes known to regulate EMT by downregulating E-cadherin in various cancer cell lines and are known targets of Smad complexes [17].

TGF-β works in concert with several other signaling pathways such as those of Ras/MAPK, Wnt/β-catenin, and NF-κB to induce EMT [18,19]. It has been shown that oncogenic Ras (Ras^V12^) signaling alters TGF-β response by inhibiting the Smad4 pathway, effectively promoting tumor invasiveness in EMT [20]. The Ras/MAPK pathway also increases cell proliferation of epithelial cells and, thus, provides a premalignant environment for EMT to occur [21]. Wnt signaling is greatly involved in cancer related EMT. Wnt signaling promotes the translocation of β-catenin, which, in turn, transcriptionally activates Wnt target genes, including cyclin D1, MMP-7, Twist, and Snail, resulting in the downregulation of E-cadherin [22,23]. E-cadherin suppresses β-catenin by forming a complex and preventing its nuclear translocation [24]. Moreover, the negative regulation of E-cadherin effectively accelerates Wnt-β/catenin-induced EMT. As a major player of inflammatory responses, NF-κB is also heavily implicated in EMT. Inflammatory cytokines, regulated by NF-κB, are primary drivers of EMT by activating transcription factors including Smads, Snail, Twist, and Zeb [25]. NF-κB also has significant crosstalk with the IL-6/STAT3 signaling pathway, which has been shown to increase MMP secretion and induce EMT in malignant cells [26,27,28]. Additionally, NF-κB has also been shown to promote Snail expression through Akt signaling [29].

MicroRNAs (miRNAs) are a class of small, endogenous non-coding RNAs that control gene expression by binding to the 3′ UTR (untranslated region) of target messenger RNAs, resulting in subsequent mRNA degradation and gene silencing [30]. Extensive research in the past has shown that each miRNA is capable of targeting multiple mRNAs from either tumor suppressors or oncogenes, and thus, miRNAs have a significant impact on carcinogenesis [31]. For example, miR-200, miR-30, and miR-34 families regulate EMT through modulating the expression of ZEB1, SNAI1, and SNAI2 genes. Conversely, certain oncogenes and tumor suppressors can also regulate miRNA expression. For example, NF-κB suppresses the expression of miR-448 in MCF7 cells, thus functioning to promote EMT induced by chemotherapy [32].

Metals and metalloids are important classes of environmental toxicants, as they are among the top substances of concern on ATSDR’s 2019 Substance Priority List (ATSDR, 2019). Toxic metal(loid)s are ubiquitous and often persist in the environment because they are natural components of the earth’s crust [33]. Humans are exposed to toxic metal(loid)s from their environment routinely. Many poor and undeveloped communities are disproportionally affected by toxic metal(loid)s due to industrial practices [34]. According to the International Agency for Research on Cancer (IARC), certain toxic metal(loid)s, such as arsenic, cadmium, chromium, and nickel, are known to cause cancer in humans [35]. Recent studies have shown that exposures to toxic metal(loid)s significantly affects the expression of miRNAs, leading to alterations in metabolism and signaling, as well as cell proliferation and differentiation. In this review, we aim to summarize the gene–miRNA–metal interactions that perturb EMT.

## 2. Arsenic

Arsenic is the number one contaminant of concern in the world. In fact, millions of people globally are impacted by arsenic through environmental and occupational exposure. A major contributor to exposure is through arsenic-contaminated water sources and soils, which are heavily relied on for drinking water, cooking, and food products [36]. Although arsenic exposure is a global issue, developing nations are disproportionately affected due to limited economic resources to remove arsenic from contaminated sources [37].

Both arsenic and inorganic arsenic compounds are classified as a Group I human carcinogen by IARC and have been associated with skin, lung, and bladder cancer [38]. Environmental arsenic exists in inorganic species, arsenate and arsenite. When inorganic arsenic is metabolized in the body, it undergoes methylation in the liver, resulting in monomethyl or dimethyl arsenic compounds [39]. Arsenic impacts multiple intracellular signal transduction pathways including Ras/MAPK and PI3K/Akt [40,41] and affects cell cycle checkpoint as well [42].

Although environmental arsenic exposure has been historically linked to human cancers, arsenic trioxide (ATO) has been used as a chemotherapeutic agent to treat acute promyelocytic leukemia [43]. In fact, ATO has been shown to inhibit DNA methyltransferase activity, reversing the methylation state of the CDH1 promoter to restore E-cadherin gene expression [44]. Recently, miRNA dysregulation has been proposed as a mechanism of action for arsenic carcinogenesis and chemotherapeutic effect.

### 2.1. Tumor Suppressing miRNAs Impacted by Arsenic Exposure

Arsenic exposure has been shown to downregulate tumor suppressor miRNAs and induce EMT. The well-studied miR-200 miRNA family has been shown to be downregulated in arsenic-treated bronchial and bladder epithelial cell lines and acquired mesenchymal phenotypes [45,46]. The miR-200 miRNA family is well-known to have direct regulation of ZEB1 and ZEB2. In bladder epithelial cell lines, MAPK/PI3K/AKT signaling was found to be dysregulated in arsenic-induced EMT [41]. Moreover, p-AKT, cyclin D3, and m-TOR were overexpressed [45]. Arsenic-transformed bronchial epithelial cells have been shown to induce the activation and nuclear translocation of β-catenin, resulting in VEGF expression. Stable expression of miR-200b reduced β-catenin target gene expression and restored its epithelial phenotype [46]. Although not directly regulated by arsenic, miR-100 plays a tumor suppressive role in arsenic carcinogenesis in BEAS-2B cells. MiR-100 has been shown to suppress the expression of several oncogenes, including IGF1R-β, CDC25A, and mTOR, and is a promising therapeutic miRNA in treating arsenic carcinogenesis [47,48].

### 2.2. Oncogenic miRNAs Affected by Arsenic Exposure

miR-21 is an oncomiR implicated in arsenic-induced EMT. One of the targets of miR-21 is programmed cell death protein 4 (PDCD4). PDCD4 expression is reduced in many tumor cells and plays a role in apoptosis, although the exact mechanism is not well elucidated [49]. In human bronchial cells, arsenite reduced PDCD4 protein and increased Twist1. Moreover, inhibiting miR-21 decreased cell migration and invasion [50]. The role in miR-21 is suggested to be directly regulated by the IL-6/STAT3 pathway [51]. This signaling pathway has been shown to be involved in arsenic-transformed human bronchial and keratinocyte cell lines [52].

miR-191 is another oncomiR in arsenic-induced EMT. In human liver epithelial cells, miR-191 expression is upregulated upon arsenite exposure [53]. miR-191 is a target of HIF-2α, which plays a role in angiogenesis and metastasis in most solid tumors [54]. Inhibition of miR-191 decreased mesenchymal the markers N-cadherin, WT-1, and α-SMA and restored its epithelial phenotype, suggesting its role in arsenic-induced EMT.

In addition to miRNA regulation, arsenic can also regulate circular RNAs (circRNAs), which are noncoding RNAs that act as transcriptional regulators and miRNA sponges. In arsenite-transformed HaCaT cells, circLRP6 and ZEB1 were found to be upregulated, while miR-455 was downregulated. miR-455 was shown to suppress ZEB1 expression and circLRP6 acts as a sponge for miR-455 [55]. Similarly, another circRNA, circ008913, is upregulated in arsenite-transformed HaCaT cells [56]. Circ008913 has been shown to inhibit miR-889, responsible for regulating the DAB2IP, a major inhibitor of the PI3K–Akt–mTOR pathway to activate ZEB1 expression [57].

### 2.3. MicroRNAs Impacted by Therapeutic Use of Arsenic

In hepatocellular carcinoma, ATO induces the expression of miR-491, which negatively regulates MMP-9 and inactivates EMT genes, Snail and Slug. Moreover, the knockdown of miR-491 induced EMT in hepatocellular carcinoma cell lines, highlighting a chemotherapeutic mechanism of ATO [58]. ATO and all-trans retinoic acid (ATRA) are used in combination as a chemotherapeutic against breast cancer. It has been shown that ATO and ATRA synergistically inhibit isomerase Pin1, effectively downregulating multiple oncogenic signaling pathways. Moreover, ATO or ATRA alone downregulate Pin1 protein and its downstream targets including β-catenin, Akt, and cyclin D1 [59,60,61]. Pin1 has also been shown to downregulate miRNA biogenesis in cancer. A microRNA analysis using 231 cells revealed that microRNAs were globally upregulated as a result of Pin1 inhibition. Western blot analysis also confirmed the downregulation of the mesenchymal markers Slug, vimentin, and ZEB-1 [62].

### 2.4. Emerging miRNA Targets in Arsenic Treated Cells

Recent studies have identified new miRNA targets in arsenic carcinogenesis. A miRNA microarray study using arsenite-transformed cells found that miR-33b was downregulated [63]. miR-33b is a tumor-suppressing miRNA and has been shown to bind to the 3′-UTR of ZEB1, inhibiting its expression, and suppressing Wnt/β-catenin signaling [64]. Arsenite exposure induced EMT in human bronchial epithelial cells and reduced levels of the miR-200 family. miR-200 depletion in arsenite-exposed cells involved the induction of ZEB1 and ZEB2 and increased methylation of miR-200 promoters. Moreover, miR-200b stable expressed cells were able to reverse and prevent arsenic-induced EMT [65]. Arsenate has been found to activate miR-124-3p and miR-16-5p in murine cranial neural crest cells [66]. miR-124-3p binds to the 3′ UTR of arrestin domain containing 1 (ARRDC1), a protein involved in vesicular trafficking and EMT in hepatocellular carcinoma [67]. miR-16-5p negatively regulates insulin growth factor 1 receptor (IGF1R), a tyrosine kinase that activates oncogenic signaling pathways, including PI3K/Akt and MAPK, and is involved in EMT seen in hepatocellular carcinoma [68].

## 3. Cadmium

Cadmium (Cd) is often used in steel manufacturing, coloring glass, and stabilizing plastic, and is a concern in occupational exposure [69]. Cd is also a major environmental pollutant, with 70% of cadmium waste coming from construction, shipbuilding, and mining [70]. Other common sources of exposure include tobacco smoke and certain foods such as shellfish, cereals, and vegetables [71]. IARC classifies cadmium and cadmium compounds as Group I human carcinogens. Epidemiology studies on Cd-exposed workers have shown that cadmium is a major cancer risk and is often linked with lung, prostate, and breast cancer. Mechanistic studies have shown that Cd affects DNA repair, promotes generation of ROS, and induces chromosomal aberrations [72].

### 3.1. Tumor Suppressing miRNAs in Cd-Induced EMT

The miR-30 family inhibits EMT by downregulating ZEB1 in lung epithelial cell lines. Cd exposure has been shown to downregulate several miRNAs of the miR-30 family [73]. As a result, mesenchymal markers, ZEB1 and vimentin, were upregulated in Cd-treated lung epithelial cell lines. Another study found that in Cd-related chronic obstructive pulmonary disease patients, serum miR-30 levels were significantly lower compared to control. Moreover, circulatory Cd was positively associated with pulmonary EMT [74].

miR-224-5p has a tumor suppressive role. It regulates the RNA-binding protein quaking (QKI), leading to miRNA stabilization and alternative splicing. It has been shown that QKI acts a tumor suppressor in different cancers by regulating EMT [75]. In BEAS-2B cells, Cd induced a downregulation of Circ-SHPRH which acts as a sponge for miR-224-5p. The downregulation of miR-224-5p and QKI resulted in a mesenchymal phenotype [76].

### 3.2. Oncogenic miRNAs Impacted by Cd Exposure

In Cd-induced pancreatic ductal adenocarcinoma, miR-221 and miR-155 were upregulated, resulting in enhanced expression of SNAIL and ZEB1 proteins, whereas miR-126, Wnt-11, and E-cadherin were downregulated [77], suggesting a positive role of miR-221 and miR-155 in Cd-induced EMT and carcinogenesis.

Key cell signaling pathways including the Ras/MAPK signaling axis are involved in Cd-mediated EMT. KRas is the most frequently mutated isoform making up 86% of all Ras mutant cancers. KRas mutations allow for constitutive Ras signaling, promoting cell survival and malignant growth. We have previously shown that KRas and its interaction partner RADIL play an important role in cell migration, invasion, and EMT [78,79]. It has also been shown that KRas, along with YAP1, coregulate FOS transcription factor to initiate EMT [80]. Inhibiting downstream targets of KRas, such as MEK1 or AKT, has been shown to reverse KRas-induced EMT [81]. Other Ras isoforms, HRas and NRas, also have EMT capabilities in colon and mammary epithelial cells, respectively [82,83].

**Figure 1:**
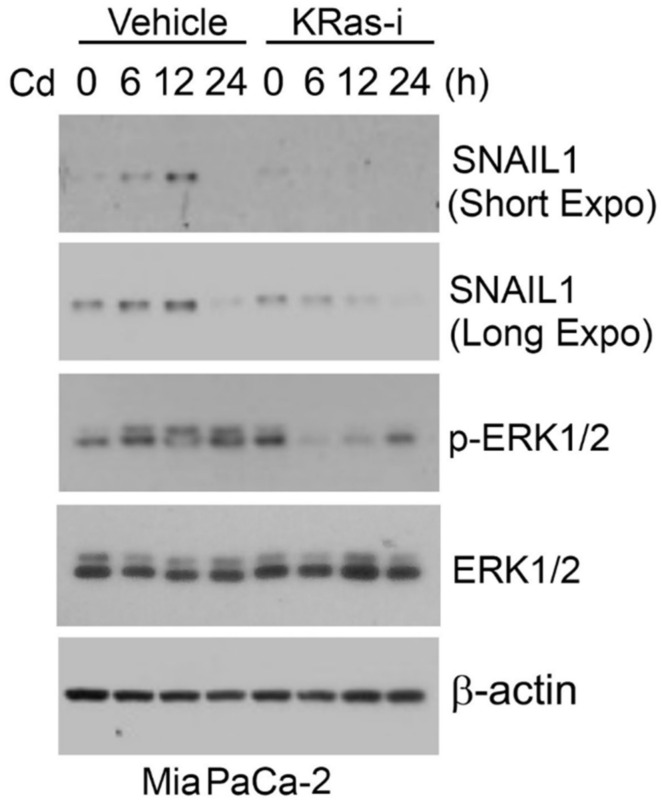
Cadmium induces expression of SNAIL1. MiaPaCa-2 cells were treated with cadmium chloride (10 μM) in the presence or absence of a specific KRas^G12C^ inhibitor (ARS-1620) for various times as indicated. Equal amounts of cell lysates were blotted for SNAIL1, phospho-ERK1/2, total ERK1/2, and β-actin. For original Western Blot, see Figure S1.

Ras/MAPK signaling plays a significant role in Cd-induced EMT. We recently showed that Cd induced SNAIL1 expression in pancreatic MiaPaCa-2 cells (Figure 1). Since MiaPaCa-2 cells harbor KRas^G12C^ mutation, we treated these cells with a specific KRas inhibitor (ARS-1620). We observed that inhibition of KRas greatly suppressed induction of SNAIL1 expression by Cd, consistent with the involvement of the RAS/MAPK signaling pathway in the induction of EMT.

YYI, a transcription factor regulating multiple cellular processes, including cell survival, division, differentiation, and apoptosis [84,85], is a downstream target of KRas. In lung cancer cells, knocking down YY1 greatly suppressed cell migration, proliferation, and angiogenesis mediated by oncogenic KRas (KRas^G13V^) [86]. In pancreatic cancer cells, oncogenic KRas activated NF-κB signaling and upregulated the expression of YY1, which, in turn, led to the repression of miR-489, an miRNA important for inhibiting migration, invasion, and colonization of transformed cells [87]. EZH2 frequently functions as a partner of YY1 in silencing miRNAs in various cancer models [88] and the oncoprotein binding domain of YY1 is crucial for its interaction with EZH2 [89]. In prostatic cancer cells, YY1 depletion reduced cell viability, coupled with increased apoptosis, which is associated with increased expression of miR-146a [88].

## 4. Nickel

Metallic nickel (Ni) is classified as Group IIB, or possibly carcinogenic to humans, whereas Ni compounds are Group I carcinogens. Ni is used in corrosion resistant alloys for iron plating and brass. Humans are commonly exposed through diet, as Ni is an essential metal in plants [90]. Other sources of exposure include pollution from mining, fossil fuels, and tobacco smoke. Epidemiology studies have found that sulfidic Ni refinery workers have a significantly higher risk of developing cancer [91].

Mechanistic studies have shown that Ni exposure induced a gain-of-function mutation in the KRas gene at codon 12 and a loss-of-function in the p53 tumor suppressor gene [92]. Additionally, Ni activates hypoxia-inducible genes through the induction of the HIF-1 transcription factor, which is commonly dysregulated in solid tumors [93]. Ni also induces the transcriptional activation of NF-κB, resulting cytokine dysregulation and allergenic effects associated with Ni-exposure [94].

### 4.1. Tumor Suppressing miRNAs Regulated by Ni

Nickel has been shown to induce loss of the repressive histone modification H3K27me3 at the ZEB1 promoter causing its upregulation. Moreover, nickel represses miR-200/205, negative regulators of ZEB1, which contributes to the upregulation of ZEB1 [95] (PMID: 34058338). Similarly, NiCl_2_ treatment induces methylation of the promoter of CDH1, thus silencing E-cadherin expression in BEAS-2B cells [96].

### 4.2. Oncogenic miRNAs Regulated by Ni

Ni-treated EGFR-mutated lung cancer cells have been shown to induce miR-21, promoting an invasive phenotype [97]. Similarly, mice treated with Nano-Ni had increased miR-21 expression and increased proinflammatory cytokines IL-6 and TNF-alpha through the NF-kB pathway. miR-4417 increases in Ni-treated BEAS-2B cells, which resulted in decreased expression of the miRNA target, TAB2. TAB2 regulates fibronectin expression, which serves as a scaffold for fibrillar ECM [98]. Moreover, miR-4417 levels did not affect E-cadherin expression but still induced EMT, which suggests that miR-4417 controls ECM breakdown through TAB2 downregulation, promoting cancer invasion [96].

## 5. Chromium

Chromium exists in two valent states: trivalent chromium [Cr(III)] and hexavalent chromium Cr(VI), with Cr(VI) being more toxic. Cr(III) is an IARC Group III carcinogen, or not classifiable as carcinogenic to humans. Cr(III) is commonly supplemented in athletic drinks and supplements because it is believed to enhance muscle growth and binds insulin, but the mechanism and risk–benefit remains heavily debated [99]. On the other hand, Cr(VI) is an IARC Group I carcinogen. Cr(VI) is used in paint, metalworks, and tanning industries, making occupational exposure a significant contributor to environmental health risk [100].

Cr(VI) induces carcinogenesis through metabolic reduction in the cell. These Cr-reduced metabolites induce genetic lesions by adducting to both DNA and proteins. Cr metabolites have also been shown to induce DNA stand breaks, DNA interstrand crosslinks, and chromosomal instability [101,102]. Cr(VI) has been suggested to activate ERK signaling and induce cell proliferation [103,104]. Research efforts are being made to look at its potential activity in deregulating EMT.

### 5.1. Tumor Suppressing miRNAs Impacted by Cr(VI)

Cr(VI) has been shown to decrease miR-27a/b, resulting in increased tumorigenesis, cell proliferation, and invasion in BEAS-2B cells [105]. Mir-27a/b was found to be a target of the Keap1/Nrf2 pathway, which recently has been shown to drive a partial EMT phenotype in non-small cell lung cancer cells and bladder cancer cells [106]. Additionally, Keap1/Nrf2 has also been shown to induce EMT in A549 cells through the activation of TGF-β via Notch signaling [107].

### 5.2. Oncogenic miRNAs Impacted by Cr(VI)

mir-21 is upregulated in Cr(VI)-treated BEAS-2B cells and downregulated PDCD4 and E-cadherin. Cr(VI) also induced secretion of IL-6 and transcriptional activated STAT3, which was found at the promoter region of miR-21. Taken together, these results suggest that Cr(VI) exposure triggers EMT through the induction of miR-21 through the IL-6/STAT-3 pathway [108].

## 6. Conclusions

**Figure 2:**
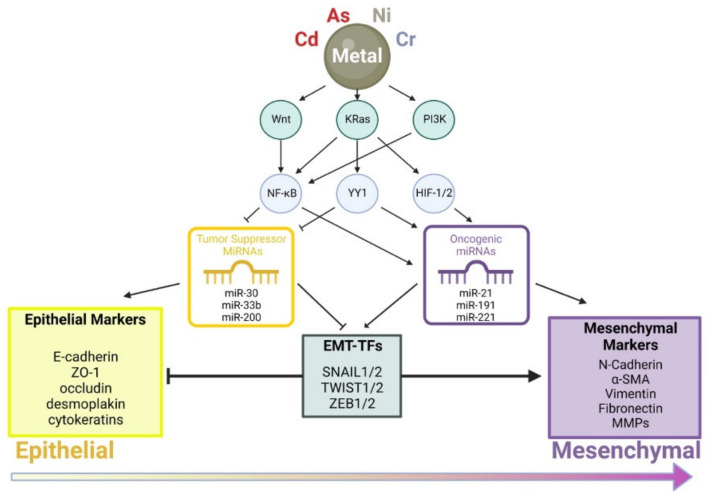
A model depicting modes of action of various toxic metal(loid)s in promoting the epithelial–mesenchymal transition.

In conclusion, toxic metal(loid)s exert a profound effect on cell migration, invasion, and differentiation through perturbing cellular regulatory processes including Wnt, Ras/MAPK, and PI3K signaling pathways (Figure 2). Alterations in cell signaling modulate activities of transcription factors, such as NF-κB, YY1, and HIF-1/2, leading to enhanced expression of oncogenic miRNAs and/or decreased expression of tumor suppressing miRNAs. These molecular changes eventually lead to the activation of master transcription factors including SNAIL1/2, TWIST1/2, and ZEB1 that drive cells toward the mesenchymal cell phenotype.

MiRNAs are a group of molecular regulators that are important to the regulation of EMT and metal carcinogenesis. However, more studies are needed to characterize lesser-known miRNA species and understand their modes of action. We also need to realize that most studies so far are limited to in vitro cell models. Understanding the complex process of how metal contaminants induce carcinogenesis through the perturbation of miRNAs and EMT in vivo will lead to the identification of new miRNA-based targets for cancer drug designs.

## Supplementary Materials

The following supporting information can be downloaded at: https://www.mdpi.com/article/10.3390/cancers14235818/s1, Figure S1: original western blots for Figure 1.
