# Supplementary figures and images for: MicroRNA–Gene Interactions Impacted by Toxic Metal(oid)s during EMT and Carcinogenesis
Source: Cancers (Basel). 2022 Nov 25;14(23):5818. doi: 10.3390/cancers14235818 (PMC9741118; doi:10.3390/cancers14235818)

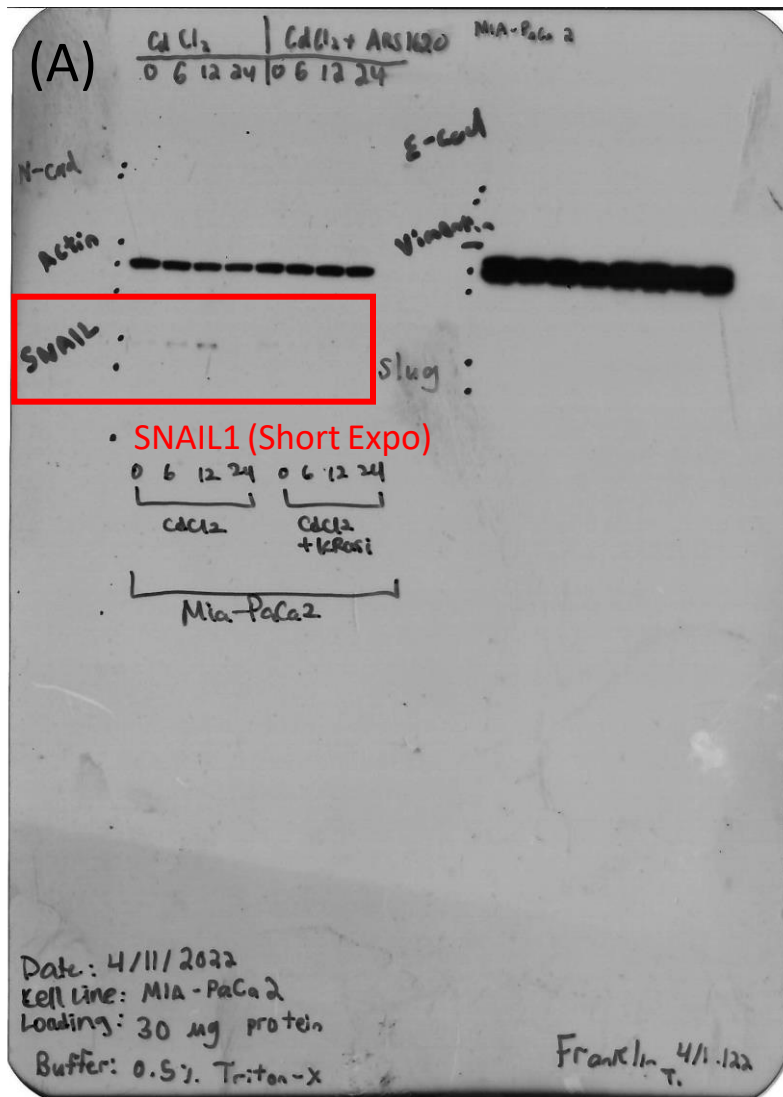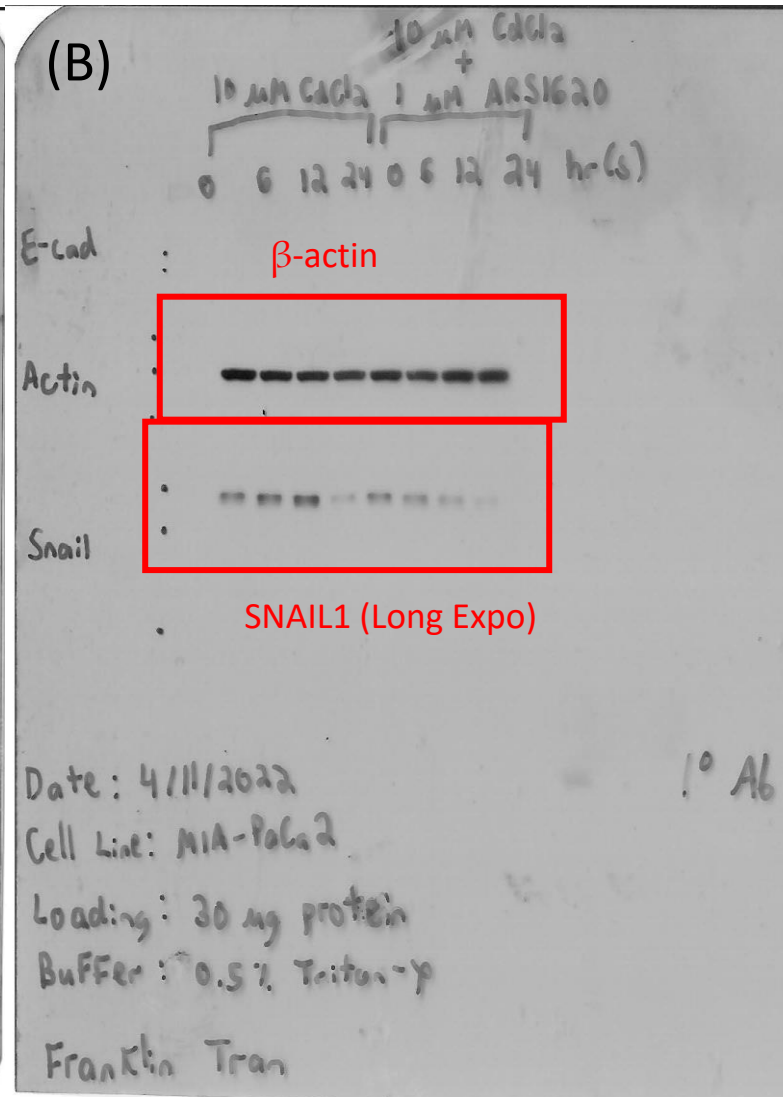

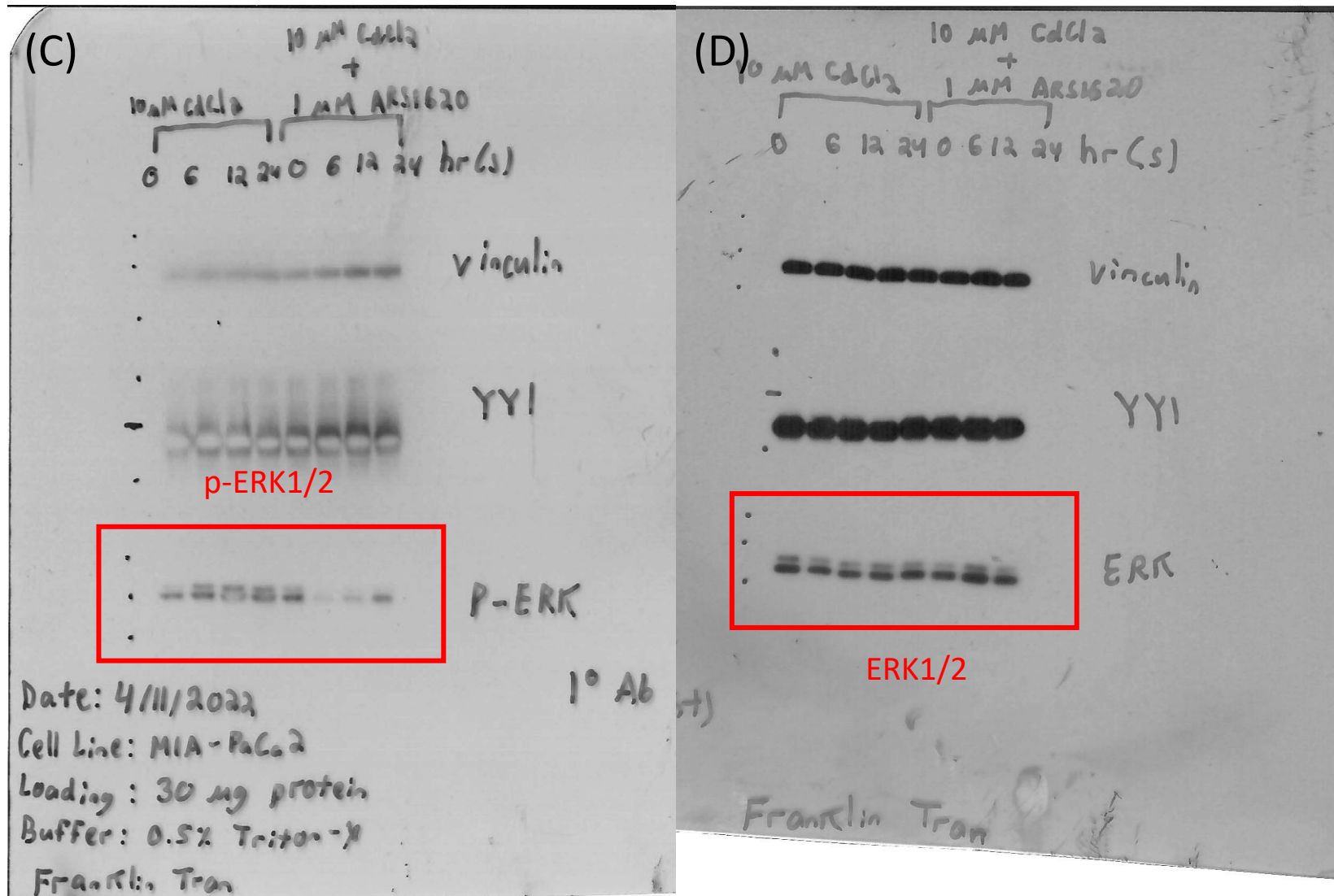

Figure S1. Western Blots for Figure 1. (A) SNAIL1 (Short Expo). (B)  $\beta$ -actin; SNAIL1 (Long Expo). (C) p-ERK1/2. (D) ERK1/2

Supplement: Supplementary file 1 [file cancers-14-05818-s001.zip › cancers-1991585-supplementary.pdf]
